# Strong correlation of gene counts and differentially expressed genes between a 3′ RNA-Seq and an RNA hybridization platform in transcriptome analyses from canine archival tissues

**DOI:** 10.3389/fvets.2025.1601306

**Published:** 2025-06-30

**Authors:** Alexander F. H. Haake, Alina K. Loriani Fard, Vladimir M. Jovanovic, Sandro Andreotti, Achim D. Gruber

**Affiliations:** ^1^Department of Veterinary Medicine, Institute of Veterinary Pathology, Freie Universität Berlin, Berlin, Germany; ^2^Department of Mathematics and Computer Science, Bioinformatics Solution Center, Freie Universität Berlin, Berlin, Germany; ^3^Human Biology and Primate Evolution, Institute of Biology, Freie Universität Berlin, Berlin, Germany

**Keywords:** dog, FFPE, nCounter, pathology, QuantSeq 3′, oncology, veterinary

## Abstract

Analyses of nucleic acids from archival tissues offer invaluable prospects for numerous fields of veterinary medicine, such as the study of differential gene expression in rare or historic diseases. The establishment of modern methodologies, however, raises questions regarding the comparability and reproducibility of data obtained from unlike tools. 3′ RNA-Seq and direct RNA hybridization are such conceptually different approaches for high-throughput transcriptome analysis. Since both are applicable to short, partially degraded mRNA fragments, they in principle allow investigations of formalin-fixed, paraffin-embedded (FFPE) tissues that are abundantly available in pathology archives. Here, we compared the two methods in several relevant details using the RNA from the same set of 35 FFPE canine tumors as input, including sample- and gene-wise count levels, gene expression strengths and directions, as well as the overlaps of differentially expressed genes (DEGs). Both methods proved suitable for their use on archival tissues with moderately to very strong overall count correlations, as indicated by a range of Pearson and Spearman means between 0.66 and 0.87. Of note, the gene-wise count correlations depended on gene expression strength. In an entity-contrasting comparison, expression directions correlated very strongly ranging from 0.88 to 0.91, but DEGs overlapped only moderately with a Jaccard index of 0.53. Finally, we contrasted the different practically relevant aspects of the two technologies with their distinct advantages that depend on the objectives and design of the study. This comparison will guide and help to select the appropriate method and to validate and interpret the data obtained.

## 1 Introduction

Research and diagnostic institutions usually store formalin-fixed, paraffin-embedded (FFPE) tissues in archives, accumulating large numbers and variations of specific diseases, samples of rare entities, or otherwise valuable properties. Such archives represent a priceless resource for retrospective biomedical studies allowing basic and applied research in many fields, including comparative oncology. Recent technologies with different conceptual approaches (1–3) have made it possible to quantify specific RNA sequences from such samples that allow a deeper insight into the dynamics of disease-specific gene expression levels and gene regulation.

However, FFPE specimens pose particular challenges in terms of RNA quality and detectability, such as contamination with RNases and other inhibitory proteins (3, 4) or diverse chemical alterations (5). The latter include RNA degradation prior to fixation, chemical modification via cross-linking with peptides by formaldehyde, and fragmentation by high temperatures during paraffin embedding and prolonged storage (6–8).

Recently developed 3′ RNA-Seq methods, such as QuantSeq 3′, generate libraries from one sequence per transcript by capturing and sequencing short fragments at the 3′ end of polyadenylated RNA (9, 10), thereby requiring significantly fewer reads compared to conventional RNA-Seq. Moreover, poly(A) enrichment, rRNA depletion, and RNA fragmentation before reverse transcription are no longer needed which simplifies and accelerates processing. Additionally, transcript length bias usually seen in traditional next generation sequencing (NGS) techniques (11) where long transcripts are artificially overrepresented (12) is circumvented, facilitating bioinformatic processing. Moreover, by capturing only short sequences of 60–80 nucleotides (nt) from the 3′ end of mRNA, QuantSeq 3′ can be used for analyzing partially degraded RNA from FFPE samples.

Alternatively, RNA hybridization panels can be employed for the analyses of specific groups of genes involved in specific disease processes. nCounter® (13) represents such a technology, employing a panel of color-coded molecular barcodes to target a pre-selected set of RNA molecules for digital quantification without prior cDNA synthesis or amplification, as opposed to RNA-Seq methods. Each unique transcript target is hybridized to a capture and reporter probe pair to generate a target-probe-complex, yielding a single count per transcript (14). This assay currently allows the detection of 800 pre-selected plus 6–55 optional, user-defined transcripts. The preselection of targeted gene transcripts is either based on commercial gene expression panels, such as select aspects in oncology or immunology, or customizable for individual research endeavors. Importantly, even partially degraded RNA may serve as adequate input.

Despite overlapping applications, several decisive differences distinguish the two methods both in study goal and practical perspectives, which are summarized in Table 1. Depending on the goal and design of a study, the two methods offer different strengths and limitations. The QuantSeq 3′ system is particularly suitable for investigating entire pathways over virtually all cellular functions. Thus, the aim is to obtain a holistic overview of the differences between contrasting groups, or in studies that focus on, for example, biomarker identification. On the other hand, the nCounter® Canine IO Panel is suitable for studies that focus directly on immuno-oncological questions in dogs, including inflammatory subtypes of cancer and clinical research involving, for example, the testing of treatment effects (15–17). The major advantage of this assay is that even lowly expressed genes can be detected with a high sensitivity as a result of the targeted analysis, whereas in a whole transcriptome approach these genes are potentially not detected due to the much higher abundance of other genes. On the other hand, prior knowledge about the genes of interest or splice variants of the transcripts is necessary to generate specific nCounter® probes, thus limiting the discovery of new or unexpected transcripts. Furthermore, it is not always possible to differentiate between different known splice variants (18, 19). However, the same applies to the QuantSeq 3′ method, since a small number of nucleotides is used to infer the presence of transcripts.

**Table 1:** Overview of conceptual and practical differences between the QuantSeq 3′ and nCounter^®^ Canine IO Panel technologies.

	QuantSeq 3′	Counter^®^ Canine IO Panel
Approach	Mostly hypothesis-generating	Usually hypothesis-driven (immuno-oncological landscape of canine tumors)
Output	Whole transcriptome (dependent on reading depth)	800 preselected immuno-oncologically relevant genes, optional 6–55 used-defined genes
Principle of method	Strand-specific NGS libraries close to 3′ end of polyadenylated RNA One fragment per transcript Direct linking of number of reads mapping to a gene to its expression	Direct quantification of specific mRNA molecules using capture and reporter probe
RNA input	0.5–500 ng	1–100 ng
Consumables and reagents	QuantSeq 3′ mRNA-Seq Library Kit FDW for Illumina® or Ion Torrent™	nCounter® IO Canine Panel (XT CodeSets) nCounter® IO Canine Primer Pool nCounter® Master Kit (including cartridges)
Workflow	Library generation with oligo(dT) primers Library amplification NGS sequencing with customized sequencing primer (or T-fill reaction) Data processing	Hybridization of RNA samples with probes over night Load onto nCounter® analysis system Data import into and analysis with nSolver™ software
Data processing	Bioinformatic data analysis pipeline necessary (raw data processing, alignment to reference genome, software for differential gene expression analysis)	Autonomous evaluation using nSolver™ with Advanced Analysis plug-in
Laboratory equipment	Sequencer (Illumina® or Ion Torrent™)	nCounter® analysis system (SPRINT, Pro or MAX/FLEX)
Bioinformatic services	Complete workflow can be ordered from the company	None (but technical support for nSolver™ is available)
Required time	4.5 h with less than 2 h hands-on time	~15 min hands-on time, overnight hybridization
Suitable for	FF, FFPE, cell lysates	Any sample type (FFPE, FF, blood, etc.)
Multiplexing	Illumina® libraries: up to 96 external barcodes Ion Torrent™ libraries: 24 in-line barcodes	12 reactions per cartridge (in a single run)

FF, fresh frozen; FFPE, formalin-fixed, paraffin-embedded; NGS, next-generation sequencing; (m)RNA, (messenger) ribonucleic acid; FDW, forward; IO, immuno-oncological.

Several independent studies have shown that both QuantSeq 3′ (9, 20) and nCounter® (21–23) are suitable for gene expression profiling performed on partially degraded RNA isolated from FFPE tissues. In a comparison of nine different methods, both nCounter® and QuantSeq 3′ performed well on up to 15 year-old FFPE tissues (24). In addition, molecular subtypes of specific tumors have been accurately identified with both approaches from FFPE tissue samples (10, 25–28).

So far, however, the data output of these two technologies has not been compared to one another in terms of mutual validation and reproducibility when using RNA from canine archival tissues as input. A comparison of the two platforms’ performances on canine FFPE tumor tissue is particularly interesting, as the dog is considered a valuable model for naturally occurring cancers in humans and other comparative oncological studies (29–31).

Therefore, this study was designed to determine the correlation of gene expression data generated by QuantSeq 3′ and the nCounter® Canine IO Panel from three types of canine tumors. In order to exploit the full potential of the immuno-oncological emphasis of this nCounter® panel, two different oncological perspectives were chosen that included a strong immunological component. First, a stage-dependent comparison of early and late stages of a tumor with stereotypical spontaneous regression was selected to compare the performance on similar tissues. For this intra-tumor comparison, canine cutaneous histiocytomas (CCH) were employed. CCH is a common benign skin tumor generally of young dogs with progenitor cells to epidermal dendritic (Langerhans) cells as the cell of origin (32). CCH’s unique regression has been speculated to be due to an immune-mediated anti-tumor host response that warrants further investigation (33–37). The two stages compared here, namely group 1 and group 3 based on Cockerell & Slauson (38), represent early and late time points in the course of CCH regression: After a short period of complete absence of lymphocytes (group 1), superficial ulceration and basolateral lymphofollicular infiltration ensue (group 2), followed by progressive infiltration of the tumor by lymphocytes (group 3), and final tumor regression with a predominance of lymphocytes and coagulation necrosis of the histiocytic tumor cells (group 4) (38).

Second, an entity-contrasting comparison between the two most common glandular, perianal tumors in the dog – the hepatoid gland adenoma (HGA) and apocrine gland anal sac adenocarcinoma (AGASAC) – was elected. Despite their virtually identical site of origin, they represent both extremes on the dignity spectrum. While HGAs hardly ever develop invasive or metastatic behavior (39, 40), malignant AGASACs commonly metastasize early to regional lymph nodes (41–44). Thus, the biological differences and the different cells of origin served as another suitable setting for a technical and bioinformatic comparison of the two methodologies.

## 2 Materials and methods

### 2.1 Selection of FFPE tissue samples

A total of 35 FFPE tissue samples from 2012 to 2021 were obtained from the archive of the Institute of Veterinary Pathology. Specimens had originally been surgically excised from privately owned pet dogs and submitted for individual diagnostic and therapeutic purposes. Dog owners had given their consent and the work was ethically approved by the State Office for Health and Social Affairs, Berlin (StN 010/23). The tumors (AGASAC: *n* = 15, HGA: *n* = 10, CCH: *n* = 5 early stage, and *n* = 5 late stage) were selected based on histopathological diagnosis on hematoxylin and eosin stained slides by a board-certified veterinary pathologist. Tumor-adjacent tissue was removed to obtain largely homogeneous tumor cell masses. Further information on the individual tissue samples is provided in Supplementary Table S1.

### 2.2 RNA extraction from FFPE samples and RNA quality control

Five 10 μm FFPE scrolls were prepared from entire cross sections, collected in sterile centrifuge tubes and stored at −80°C. Total RNA was extracted using the PureLink™ FFPE Total RNA Isolation Kit (Thermo Fisher) according to the manufacturer’s guidelines. Total RNA concentrations were measured with the NanoDrop™ 2000c spectrophotometer (Thermo Fisher) and quality was determined using the Agilent 5200 Fragment Analyzer (Agilent Technologies, Inc., Santa Clara, United States) employing the DNF-471F33 - SS Total RNA 15 nt - FFPE Illumina DV200 method mode with a range of smear analysis from 200 to 20,000 nt. Only samples with a total RNA quality number (RQN) (45) of > 4 and a DV_200_ (percentage of RNA fragments over 200 nt in length) (46) of > 64.5% were chosen. For RNA quality classification, the Illumina® recommendations were applied, which denote a DV_200_ of > 70% as high and a DV_200_ of 50–70% as medium quality. For data on RNA quality, see Supplementary Table S2. Total RNA was treated with DNase I. Both the nCounter® and QuantSeq 3′ analyses were employed on the total RNA from the same isolation batches.

### 2.3 Direct mRNA hybridization

The RNA (150–250 ng) was hybridized to the nCounter® Canine IO Panel XT CodeSets (NanoString Technologies, Inc., Seattle, WA, United States), including probes representing 780 pre-selected genes and 20 “housekeeper” genes. A 30 probe Panel Plus (Supplementary Table S3) was added to the hybridization of the HGA and AGASAC samples following the manufacturer’s hybridization protocols (manual IDs: MAN-10023-11, MAN-10056-06). This Panel Plus included genes of further interest for this entity comparison. Hybridized samples were loaded onto the nCounter® MAX Analysis System’s Prep Station (NanoString) for purification and immobilization on sample cartridges, transferred to the Digital Analyzer for data collection and analyzed following the manufacturer’s user manual (manual ID: MAN-C0035-08).

Following the workflow described in the manufacturer’s recommendations (manual IDs: MAN-C0019-08, MAN-C0011-04), the reporter library files (RLF) and reporter code count (RCC) files were imported into the nSolver™ 4.0 Analysis Software (NanoString). Quality control and normalization followed default settings. Differential gene expression (DGE) analysis was implemented with the R 3.3.2-based Advanced Analysis 2.0 plug-in (version 2.0.134) with the recommended statistical settings. The raw, normalized, and DGE data were exported and read into R / Python Jupyter notebooks for further bioinformatic analysis and correlation calculations.

### 2.4 3′ RNA sequencing

The RNA was sequenced with QuantSeq 3′ (Lexogen GmbH, Vienna, Austria) at Lexogen Services. DNase I treated total RNA (500–1,000 ng from HGA and AGASAC; 375 ng from the early and late CCH stages) was processed with the QuantSeq 3’ mRNA-Seq FWD Library Preparation Kit (Lexogen) according to the manufacturer’s guidelines (user guide: 015UG009V0251) using the low-quality RNA protocol. Quality of the libraries was determined with the Agilent 5300 Fragment Analyzer (DNF-474-33 - HS NGS Fragment 1-6000 bp method mode). The samples were pooled in equimolar ratios. The library pool was quantified using a Qubit™ dsDNA HS assay kit (Thermo Fisher) and sequenced utilizing an Illumina® NextSeq™ 500 system with a SR75 High Output Kit. In total, 0.011 sequencing lanes were used per sample, resulting in an output of 4,025,165 to 7,548,231 trimmed reads for the HGA and AGASAC samples with an average input read length of 63.41 to 71.6 nt and 5,426,266 to 7,783,141 trimmed reads for the CCH samples with an average input length of 66.21 to 71.65 nt.

The FASTQ sequencing files were first pre-processed (adapter trimmed, filtered) using Cutadapt (47) and subsequently aligned to the NCBI Reference Sequence (RefSeq) assembly for the dog (*Canis lupus familiaris*) CanFam3.1 (48) (GCF_000002285.3) with the STAR aligner (49). Read counts per gene were generated with two rounds of featureCounts (50) to mitigate the effect of too short 3′ UTR annotations. Reads that remained unassigned in the first round were subjected to the second round of featureCounts on an adjusted annotation with a 3′ extension by 2 kilobases. Multi-mapped reads were retained and all counts distributed evenly across all mapping locations. For comparison with nCounter® counts, the raw gene counts were normalized with the edgeR package (51–53). DGE analyses were performed with R using DESeq2 (54). The workflow is fully accessible in figshare (see data availability, DOI: 10.6084/m9.figshare.25768587). The significance thresholds were set at a log_2_ (fold change) (log_2_FC) of ≤ −1 or ≥ 1 and an adjusted p-value (p_adj_) of ≤ 0.05.

As the nCounter® Canine IO Panel probes were designed using CamFam3.1, the same reference genome for QuantSeq 3′ read alignment was used for proper gene annotation comparison, despite the dog reference genome having been updated since. This same annotation offers a more robust background for comparison, as genes have been either dropped or added in the more recent genome.

### 2.5 Biological comparisons for differential gene expression

Two biological comparisons were chosen to test whether data sets stemming from an intra- or an inter-tumor contrast might be more prone to errors, possibly reflected in a weaker correlation of results from different measurements. For the inter-tumor, entity-contrasting comparison, the HGA (*n* = 10) was compared to the AGASAC (*n* = 15 for gene correlation, *n* = 11 for DGE) as baseline (from now on “HGA versus AGASAC”) and for the intra-tumor, stage-dependent comparison, the early CCH stage (*n* = 5) was compared to the late CCH stage (*n* = 5) as baseline (from now on “CCH early versus late”).

### 2.6 Correlation calculation

**Figure 1:**
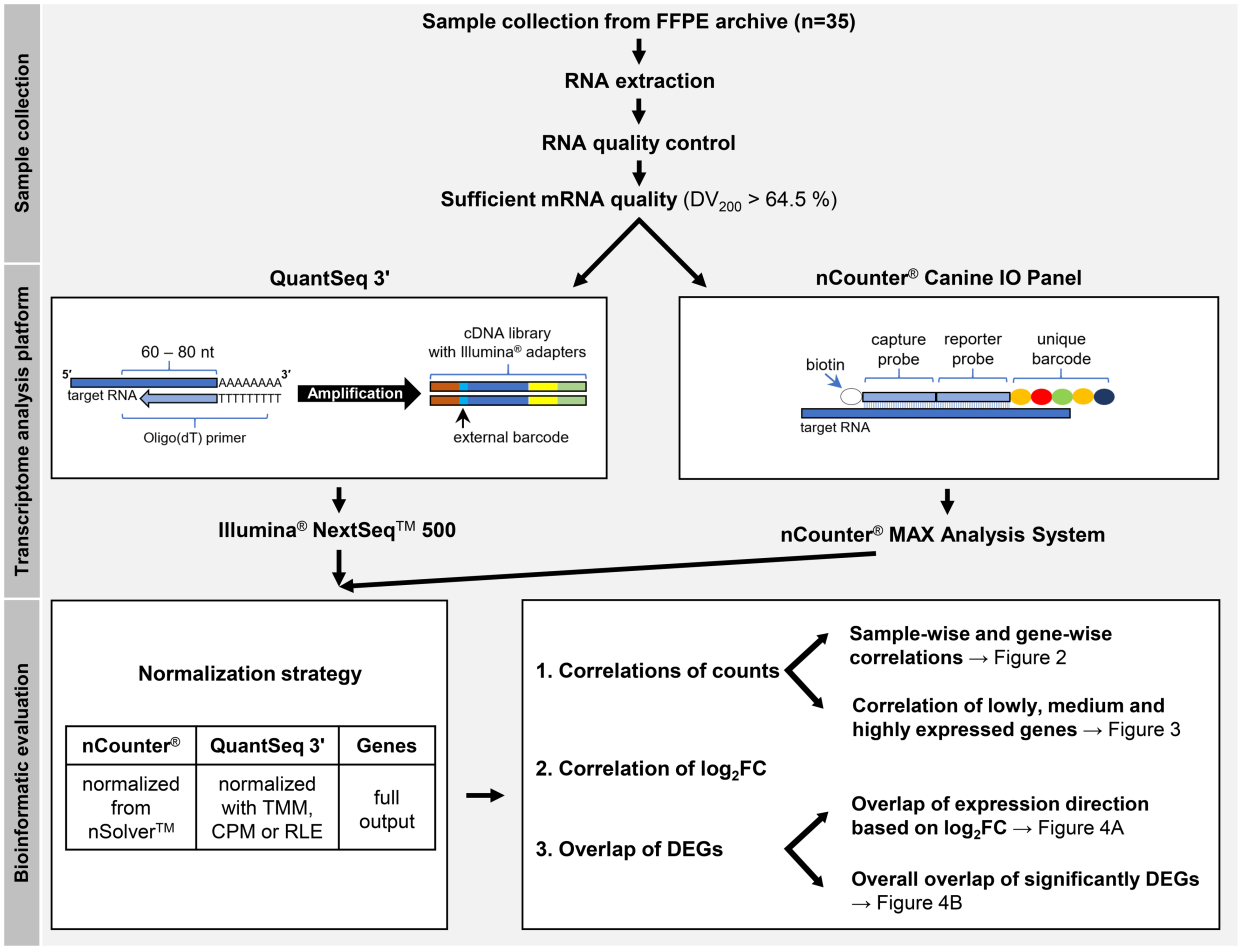
Study workflow. The schematic representations of QuantSeq 3′ and the nCounter^®^ Canine IO Panel depict the respective basic principles of the technologies. For correlation calculations of gene abundance, nCounter^®^ read counts were normalized with nSolver^™^, while QuantSeq 3′ read counts were normalized using three different methods based on the full set of known genes, not only the panel subset considered by nCounter^®^. At the level of QuantSeq 3′ data normalization, three different commonly applied normalization methods were compared. These were TMM (Trimmed Means of M-values), CPM (counts per million), and RLE (relative log expression). FFPE = formalin-fixed, paraffin-embedded, DV_200_ = percentage of RNA fragments over 200 nucleotides (nt) in length, DEG = differentially expressed genes, log_2_FC = log_2_ (fold change).

For correlation calculations of gene abundance, nCounter® read counts were normalized with nSolver™, while QuantSeq 3′ read counts were normalized using three different methods based on the full set of known genes (Figure 1). nCounter® reads were normalized in nSolver™ in a two-step process with positive control and “housekeeping” gene normalization. At the level of QuantSeq 3′ data normalization, three different commonly applied normalization methods were compared. These were TMM (Trimmed Means of M-values); the method used by edgeR, CPM (counts per million), and RLE (relative log expression); the method used by DESeq2. All three methods were implemented within the edgeR package.

Different correlation coefficients were calculated for each the correlation of counts and the differentially expressed genes (DEGs) based on their log_2_FC values. The Pearson correlation coefficient indicated the correlation of counts and Spearman’s rank correlation coefficient the correlation of gene rank. To mitigate the effect of outliers, the Pearson correlation was also calculated on log-transformed counts with a pseudocount of 1 (from now on “Pearson-log”) for the sample-wise correlation calculation.

Count correlations were considered on a sample-wise (with Pearson-log and Spearman) and gene-wise (with Pearson and Spearman) level. The sample-wise level evaluated the correlation of counts of all genes per matched FFPE sample in both analysis methods used. The gene-wise level viewed the correlation of counts of an individual gene across the analyzed sample pairs.

The interpretation of the correlation strengths was based on previous publications for application in the medical field (55, 56): ≥ 0.8 = very strong, ≥ 0.6 to < 0.8 = moderately strong, ≥ 0.3 to < 0.6 = fair, and < 0.3 = poor.

For the “HGA versus AGASAC” comparison, all 25 samples were used for the sample- and gene-wise count correlations, as a higher sample size generally improves data reliability. For DEG correlation, only the 21 primary tumor samples were included, thereby decreasing some biological variability in the tissue background within the sample cohort. All 5 CCH samples in either the early or late stage were used in both count and DEG correlations.

### 2.7 p-value adjustment

Different procedures for the calculation of the false discovery rate (FDR) used for p-value adjustment during DGE calculation are routinely used. The nSolver™ user manual recommends using the Benjamini-Yekutieli (BY) procedure (57). On the other hand, DESeq2 routinely employs the Benjamini-Hochberg (BH) procedure (58). Thus, to allow for better comparisons, the BY method was employed on all data shown here, as it is the more conservative and discriminating of the two (59–61).

## 3 Results

### 3.1 Normalization of reads from both techniques and correlation of counts

To begin, the genes included on the nCounter® Canine IO Panel were identified which are not annotated in CanFam3.1 and thus could not be routinely found in the QuantSeq 3′ data after alignment to this genome assembly. The genes were considered “missing” or without overlap between the two methods. It was consequently impossible to calculate correlation coefficients for these genes.

In the “HGA versus AGASAC” comparison, out of 830 genes included on the nCounter® panel with Panel Plus, 821 genes (98.9%) were found in the QuantSeq 3′ data. The following nine genes (1.1%) were missing in the QuantSeq 3′ data: FCAR, HAVCR2, IFNA7, IGHG, IGHM, IL2, MIF, TRAC, and TRBC.

Similarly, in the “CCH early versus late” comparison, out of 800 genes included on the nCounter® panel, ten genes (1.25%) were missing in the QuantSeq 3′ data: CCR2, FCAR, IFNA7, IGHG, IGHM, MIF, TRAC, TRBC, TRGC2, and TRGC3. Thus, 790 genes (98.75%) genes overlapped.

To determine the correlations on a count level, counts of all genes per matched FFPE sample analyzed with both nCounter® and QuantSeq 3′ (sample-wise level) were investigated. Overall, the sample-wise count correlations exhibited a narrow distribution of correlations and all correlation medians on this level were very strong in both biological comparisons (> 0.83).

**Figure 2:**
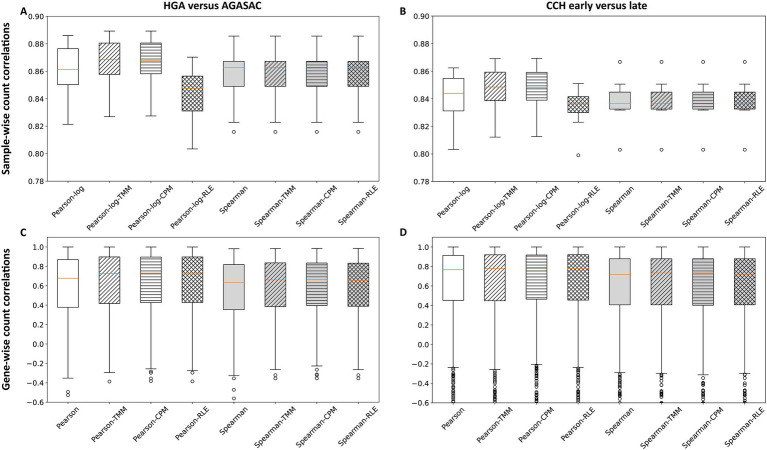
Correlation of counts from QuantSeq 3′ and the nCounter^®^ Canine IO Panel. The box plots show the sample-wise **(A,B)** and gene-wise **(C,D)** correlations of counts from both methods. Results for the entity-contrasting comparison (“HGA versus AGASAC”; **A,C**) and the stage-dependent comparison (“CCH early versus late”; **B,D**) are shown. The Pearson (for gene-wise) or Pearson-log (for sample-wise) correlation (white background) indicates the correlation at the count level, while the Spearman correlation (gray background) reflects the values at the gene rank level. The calculations were performed without normalization (patternless) and with three different normalization methods: Trimmed Means of M-values (TMM; obliquely striped), counts per million (CPM; horizontally striped), and relative log expression (RLE; reticulated). Orange horizontal line represents the median. Circles indicate outliers.

For the “HGA versus AGASAC” comparison, data obtained with TMM provided a very strong count correlation (Pearson-log median: 0.87). All normalization methods, however, similarly yielded very strong count correlations (Pearson-log medians: > 0.84). All gene rank correlations were identically very strong (Spearman medians: 0.86) among all normalization methods (Figure 2A).

In the “CCH early versus late” comparison, the utilization of TMM also provided a very strong count correlation (Pearson-log median: 0.85). The data from all normalization methods, however, likewise yielded very strong correlations (Pearson-log medians: > 0.83). All gene rank correlations were equally very strong (Spearman medians: 0.84) among all normalization methods (Figure 2B).

For the sample-wise count correlation calculations in both biological comparisons, the application of TMM and CPM (medians: 0.87 and 0.85, respectively) both slightly improved the Pearson-log correlations compared to the correlations with no normalization (medians: 0.86 and 0.84, respectively). However, the application of RLE slightly decreased the correlations (medians: 0.85 and 0.84, respectively). No improvement was seen after implementing the three normalization methods on the Spearman correlations; here all coefficients were identical (medians: 0.86 and 0.84, respectively).

In summary, all sample-wise count correlations were very strong with TMM, CPM, and RLE. Both the Pearson-log and Spearman correlations were slightly stronger in the entity-contrasting comparison (“HGA versus AGASAC”) than in the stage-dependent comparison (“CCH early versus late”).

Next, the correlations on a count level based on the counts of an individual gene across FFPE sample pairs analyzed with both nCounter® and QuantSeq 3′ (gene-wise level) were examined. Altogether, for the gene-wise count correlations, there was a much wider distribution of correlations.

Moderately strong (medians: > 0.63) correlations were calculated for the “HGA versus AGASAC” comparison (Figure 2C). This was observed on the level of counts (Pearson medians: > 0.67) and gene rank (Spearman medians: > 0.63). All normalization methods generated stronger correlations compared to no normalization (Pearson median: 0.67, Spearman median: 0.63). The application of RLE resulted in a slightly stronger correlation of counts (Pearson median: 0.732), compared to TMM (Pearson median: 0.726) and CPM (Pearson median: 0.731). With the use of TMM and CPM, almost identical gene rank correlations (Spearman median: 0.66) were calculated, compared to RLE (Spearman median: 0.65).

The “CCH early versus late” comparison also showed a much larger distribution of correlation values in all normalization methods (Figure 2D). This was observed on the level of counts (Pearson medians: > 0.76) and gene rank (Spearman medians: > 0.71). The application of all three normalization methods only slightly improved the correlations on count level (Pearson medians: > 0.77), compared to no normalization (Pearson median: 0.76). The application of CPM resulted in a slightly stronger correlation for counts (Pearson median: 0.79), compared to TMM and RLE (Pearson medians: 0.777 and 0.779, respectively).

Notably, both the Pearson and Spearman correlations were slightly stronger in the stage-dependent comparison (“CCH early versus late”) than in the entity-contrasting comparison (“HGA versus AGASAC”), contrary to the sample-wise results.

### 3.2 Gene-wise correlation by gene expression level

**Figure 3:**
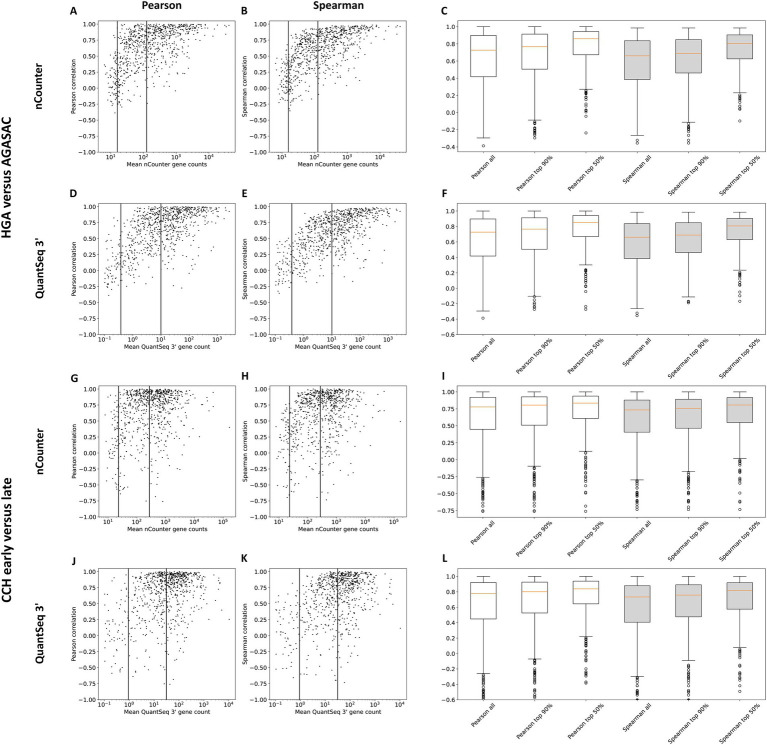
Correlation coefficients for genes with low, medium, and high mean expression. Results for the entity-contrasting comparison (“HGA versus AGASAC”; **A–F**) and the stage-dependent comparison (“CCH early versus late”; **G–L**) are shown. Scatter plots present the correlation (y-axis) of the counts of both methods plotted against the mean nCounter^®^ **(A,B,G,H)** or QuantSeq 3′ **(D,E,J,K)** log gene count (x-axis). Pearson **(A,D,G,J)** and Spearman rank **(B,E,H,K)** correlation coefficients were calculated with the TMM normalization method. The vertical lines indicate the thresholds between all, the top 90%, and top 50% of expressed genes. Box plots depict the correlations of all the genes included in these expression strength groups for nCounter^®^ **(C,I)** and QuantSeq 3′ **(F,L)**. Pearson (white) and Spearman rank (gray) correlation coefficients were calculated. Orange horizontal lines indicate medians. Circles indicate outliers.

To identify the gene-wise correlations of gene counts depending on the respective gene expression level, the Pearson and Spearman correlation coefficients normalized with TMM were plotted against the mean gene expression data from either nCounter® or QuantSeq 3′ (Figure 3). Thresholds were drawn based on the gene distribution within the scatter plots. All gene counts above a respective threshold were grouped into an expression strength category and the correlation coefficients calculated for each of the respective genes. Specifically, the mean expression correlations for all, the top 90%, and 50% of genes were calculated. In both comparisons, the median correlation among all genes including the lowly expressed genes was weaker (“HGA versus AGASAC”: Pearson: 0.73, Spearman: 0.66/“CCH early versus late”: Pearson: 0.77, Spearman: 0.73) than among all genes excluding the lowly expressed genes (“HGA versus AGASAC”: Pearson: 0.77, Spearman: 0.69/“CCH early versus late”: Pearson: 0.8, Spearman: 0.75) and including only the highly expressed genes (“HGA versus AGASAC”: Pearson: 0.85, Spearman: 0.8/“CCH early versus late”: Pearson: 0.83, Spearman: 0.81). Summarizing, the correlation of genes including the highly expressed genes was strongest.

### 3.3 Correlation of log_2_FC and differentially expressed genes

To ascertain the correlations of genes based on their differential gene expression in the two biological comparisons, it was first necessary to calculate the correlations of the log_2_FC values from DESeq2 without log_2_FC-shrinkage (log_2_FC-shrinkage = shrinks estimated effect size toward zero, stronger for genes with little information, i.e., low average read counts that can likely yield artificially high log_2_FC estimates) on the overlapping genes. For the “HGA versus AGASAC” comparison, both correlations were very strong (Pearson: 0.91, Spearman: 0.87) based only on the log_2_FC values of the genes. For the “CCH early versus late” comparison, on the other hand, both correlations were only moderately strong (Pearson: 0.68, Spearman: 0.72).

In order to determine the overlap of differential gene expression direction, the log_2_FC values from DESeq2 with shrinkage were collated into scatter plots. Therefore, the log_2_FC of a given gene from the QuantSeq 3′ data was plotted against the log_2_FC of the corresponding gene from the nCounter® data. The dots of the corresponding genes were colored according to the significance thresholds p_adj_ ≤ 0.05 and log_2_FC ≤ −1 or ≥ 1 and the correspondence of the expression direction based on the log_2_FC (≤ −1 = “down”; log_2_FC ≥ 1 = “up”).

**Figure 4:**
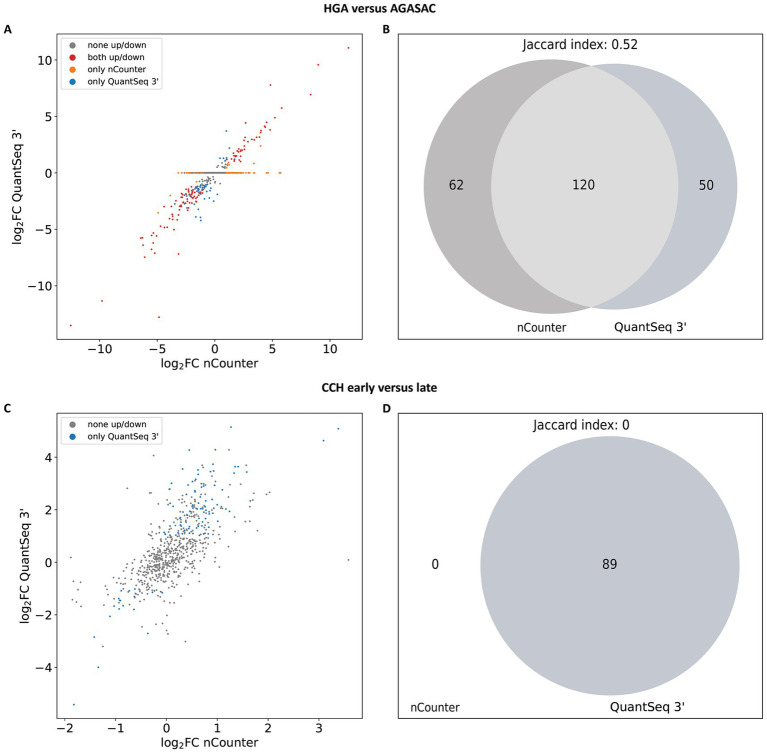
Overlap of log_2_FC direction and significantly differentially expressed genes (DEGs) from the subset of nCounter^®^ Canine IO Panel targets. **(A,B)** Results for the “HGA versus AGASAC” comparison (entity-contrasting comparison). **(C,D)** Results for the “CCH early versus late” comparison (stage-dependent comparison). **(A,C)** Scatter plots show the log_2_FC of a given gene from the QuantSeq 3′ data (y-axis) plotted against the log_2_FC of the corresponding gene from the nCounter^®^ data (x-axis). The dots are colored according to the correspondence of expression direction: Significantly (p_adj_ ≤ 0.05 and log_2_FC ≤ −1 or ≥ 1) highly/lowly expressed in both methods (red), significantly differentially expressed in only one method (nCounter^®^: orange, QuantSeq 3′: blue), or not significantly differentially expressed in both methods (gray). **(B,D)** Venn diagrams depict the overlap (light gray), if any, of significantly DEGs from nCounter^®^ and QuantSeq 3′. DEGs exclusively detected by nCounter^®^ are colored in dark gray (left), while DEGs found only in QuantSeq 3′ are labeled in slate gray (right). The p_adj_ were corrected with the Benjamini-Yekutieli (BY) method and the log_2_FC were calculated with shrinkage from DESeq2 for the scatter plot and Venn diagram.

The scatter plot for the “HGA versus AGASAC” comparison showed a total of 599 genes (Figure 4A). One hundred and twenty genes (79 “down”; 41 “up”) had the same log_2_FC direction (red dots). One hundred and twelve genes were predicted only in one of the methods: 62 in nCounter® (orange dots): 16 “down”; 46 “up” and 50 in QuantSeq 3′ (blue dots): 42 “down”; 8 “up.” No genes were classified in opposite directions. Three hundred and sixty-seven genes fell under the significance thresholds.

For the “CCH early versus late” comparison, a total of 658 genes were displayed in the scatter plot (Figure 4C). However, with a p-value threshold based on the BY adjustment, no genes were recognized by nCounter® and 89 genes (15 “down”; 74 “up”) only by QuantSeq 3′. The other 569 genes fell under the significance thresholds.

The lists of DEGs with their corresponding log_2_FC values and p_adj_ from both methods calculated by DESeq2 with shrinkage (scatter plot groups) from both biological comparisons are provided in Supplementary Table S4.

Differential gene expression (DGE) analysis performed using the BY p-value adjustment and log_2_FC with shrinkage calculated a total of 182 significantly differentially expressed genes (DEGs) for nCounter® and 170 significantly DEGs for QuantSeq 3′ in the “HGA versus AGASAC” comparison. To identify the overlap of the significantly DEGs in the “HGA versus AGASAC” comparison, a Venn diagram was created using the Matplotlib Python library (62). An overlap of 120 significantly DEGs was found in the “HGA versus AGASAC” comparison (Figure 4B), corresponding to a Jaccard index of 0.52.

In the “CCH early versus late” comparison, no significantly DEGs were calculated for nCounter® and a total of 89 significantly DEGs for QuantSeq 3′ were computed. As there were no significantly DEGs in the nCounter® data, the Venn diagram showed no overlap, matching a Jaccard index of 0 (Figure 4D).

## 4 Discussion

### 4.1 Stronger correlations compared to similar correlation studies

Here, we present the first systematic comparison between data resulting from the technical platforms QuantSeq 3′ and nCounter® using the Canine IO Panel on canine archival FFPE tissue. Previous correlation studies had contrasted data from QuantSeq 3′ or nCounter® to other mRNA quantification methods utilizing FFPE tissue. These had, for example, used different human FFPE tissues to directly compare nCounter® gene selections to other RNA-Seq methods or RT-PCR. For instance, a comparison of immune gene expression in 27 clear cell renal cell carcinoma samples between the nCounter® Pan Cancer Immune Profiling Panel and the Oncomine™ Immune Response Research Assay on FFPE specimens had revealed a moderately strong correlation (Spearman: 0.73) of fold changes for 248 shared genes. On a gene-wise level, 226 of these genes had shown positive correlations and 16 had negative correlations. The mean Pearson correlation for all genes had been fair at 0.45 (range: 0.98 to −0.25) (63). Comparing 20 genes in oral carcinoma samples using custom nCounter® CodeSets and RT-PCR, a moderately strong overall correlation (Pearson: 0.59) had been calculated (22) for gene expression levels in 19 FFPE sample pairs. In contrast, the correlation results of our study on canine tissues were stronger on both count and log_2_FC levels. This could be due to the improved compatibility of nCounter® and QuantSeq 3′ for FFPE material compared to the Oncomine™ assay and RT-PCR that had been employed in the previous studies. Considering RNA quality, the DV_200_ values of the total RNA in the samples chosen for this study on canine tissues were predominantly of high quality (n = 31 with DV_200_ > 70%) with some samples with medium quality (n = 4 with DV_200_ 50–70%). One previous study (63) had not provided RNA quality data. The other study (22) had employed only the RNA integrity number (RIN) and disclosed the use of strongly degraded RNA (mean RIN: 2.3, range 1.5–2.5). However, the DV_200_ has been shown to be a superior criterion to evaluate RNA quality compared to the widely used RIN, especially when employing partially degraded RNA from FFPE samples (46). Thus, somewhat superior RNA quality may have contributed to the slightly better correlations in our comparison.

Other studies performed on cancer cell lines (18) or peripheral blood (64) had shown that the correlations on count level are dependent on the gene expression level. Generally, weaker correlations had been observed for genes with low expression levels and stronger correlations had been calculated for genes with high expression levels. This is in line with our study’s results: both nCounter® and QuantSeq 3′ exhibited the same expression-level dependent correlation phenomenon, with stronger correlations for higher expressed genes.

The strong correlations observed in this study raise much hope for retrospective studies using samples from veterinary pathology archives, as routinely stored FFPE tissues seem principally accessible for transcriptome analyses using both nCounter® and QuantSeq 3′. In comparison to FFPE material, fresh, or fresh-frozen tissue is much more difficult to obtain in veterinary and comparative pathology for logistical, ethical, potentially infectious, and legal reasons. Additionally, the cost of RNA-Seq has greatly decreased since its first introduction and in combination with the easy storage of FFPE material provides a cost-effective research and diagnostic tool for veterinary researchers and clinicians. Especially in favor of Russell’s and Burch’s 3 R (reduce, refine, replace) principles to improve ethical concerns when using experimental animals, it is imperative to obtain the highest possible benefit out of animal tissues. Obviously, routine archiving of FFPE tissues for many decades has proven to be a fortunate circumstance.

### 4.2 Confounders and influences on transcriptome data from FFPE samples

Confounding factors when using FFPE material, however, include the type, buffering state, and concentration of formalin used, duration of fixation, tissue processing, and storage conditions, which all may impact RNA quality (1, 6, 65–68). In the case of this study, in which all tissues stemmed from routinely fixed, processed, and stored samples as part of the institutional diagnostic service, such limitations seemed to have had little, if any impact, on the results based on the RNA quality (Supplementary Table S2).

Still, multiple other aspects likely have an influence on correlations of data from different transcriptome analysis methodologies and might also account for the differences in DEGs detected in both methods in this study. To begin, the differing detection methods may result in slight discrepancies in the identification of transcripts and bioinformatic alignment of sequences after RNA-Seq may not be as specific as the probes used in nCounter®. Furthermore, by focusing on a selection of approximately 800 genes, the nCounter® panel offers the possibility to detect even weakly expressed transcripts, which may be overlooked compared to highly expressed genes, depending on the depth of sequencing in the case of QuantSeq 3′. However, pre-selected gene panels have some inherent limitations due to their focused design compared to genome-wide approaches, such as RNA-Seq. As expression analysis is restricted to specific genes of interest, changes in other genes not included on the panel remain undetected. Thus, potential novel biomarkers, broader biological processes, or unforeseen gene interactions can remain unidentified (69). Incomplete interpretation of underlying mechanisms or the oversight of key regulatory genes involved in a disease phenotype can result. Rather, panels are intended for focused research questions or validation of known targets. The choice of genes selected for inclusion is dependent on current knowledge and thus introduces a bias, restricting the scope of discovery. The gene set enrichment analysis tools available for RNA-Seq are of little statistical value for panels, as the background gene set is limited and biased by the panel’s focus. Thus, pre-selection violates the statistical assumption of unbiased, comprehensive, and genome-wide gene expression data (70).

Furthermore, different normalization methods may have an effect. This study employed the nSolver™-embedded normalization method for the nCounter® data and compared three common normalization methods (TMM, CPM, RLE) for the QuantSeq 3′ data. All methods performed similarly, with only minimal differences that seemingly did not influence the strength of correlations. Extending beyond these normalization methods, there is however a multitude of other approaches, which were developed to correct biases or weaknesses, such as accounting for gene length, refinement for FFPE RNA-Seq data, or microarray input (71, 72). For nCounter® data, further methods have likewise been developed for normalization and differential expression analysis apart from nSolver™. As the three normalization methods employed in this study provided little differences in correlations, it was not deemed necessary to test the impact of alternate normalization strategies on count correlations. Instead, it seems that DESeq2 and edgeR can reliably be used for count normalizations on QuantSeq 3′ data and that the software package one uses for normalization will likely have very little impact on the data. Similarly, other packages or approaches for differential gene expression (DGE) analysis are available (73). Again, in this study, the impact that these alternate DGE calculations may have on DEG correlations was not investigated. Overall, the analysis of DEGs is based on different approaches, i.e., statistical calculations, available software, and pathway lists for enrichment analyses, when using transcriptomic platforms. The nCounter® reads are usually fed into the nSolver™ software with the Advanced Analysis plug-in and pathway analyses are conducted with the program’s inbuilt modules. Annotations are assigned to most genes, which are provided in annotation files. The annotations for the Canine IO Panel are based on two previously developed human panels: the immune response categories originate from the PanCancer Immune Profiling Panel and the functional annotations are based on the IO 360 Panel. These annotations in turn stem from the Gene Ontology (GO) (74, 75), Kyoto Encyclopedia of Genes and Genomes (KEGG) (76), and Reactome (77, 78) databases. If an investigator has another research focus in mind, a gene curation team is able to annotate genes included on a panel with suitable terms. A custom annotation route, using an annotation engine, assigns annotations to customized gene add-ons (Panel Plus) or CodeSets directly from a database based on context (NanoString curation team, personal communication). Additionally, based on a specific research query, each investigator has the ability to manually change and adjust the annotations in an annotation file. This can lead to fluctuations and different statistical outcomes and should be transparently declared in consequent publications. With a multitude of gene annotation databases available for pathway analysis, such as GO, KEGG, Reactome, and Molecular Signatures Database (MSigDB) (79, 80), the nCounter® annotations add further nomenclature for gene grouping categories and pathways, which are not always transferable and/or interchangeable when comparing terms. However, researches may be able to answer questions more target-oriented this way.

Noteworthy, in the stage-dependent comparison conducted in this study, 89 DEGs were detected using QuantSeq 3′, but none using nCounter®. As previously outlined, it is generally to be expected that the two methods will yield divergent results, partly due to the differing scope of potentially detectable transcripts (whole transcriptome versus focus on 800 genes) and partly, to a lesser extent, due to different underlying detection methods used. Still, when comparing the stages of CCH, it must be emphasized that there are only minor differences at the transcriptome level (36). This is also reflected by the low number of detected DEGs using QuantSeq 3′ in the stage-dependent contrary to the entity-contrasting comparison in this study. However, studies that specifically had examined individual transcripts utilizing quantitative PCR found differences between early and late stages of CCH, for example in expression of proinflammatory cytokines (34). Thus, further investigation is required in order to resolve this discrepancy. Larger group sizes than those used in this study (*n* = 5) might be necessary to detect a larger number of significantly DEGs. Additionally, to the presumably minor differences at transcriptome level, the number of DEGs is likely to be reduced by the implementation of the BY procedure, which has been demonstrated to be more conservative than other FDR calculation methods (57–61).

Major differences also lie in data processing. As with all large-scale transcriptome analysis systems, vast amounts of raw data are generated requiring further bioinformatic analysis. The QuantSeq 3′ system provides nucleotide sequences in FASTQ data files for alignment and normalization using standard bioinformatic methods. Prior bioinformatic know-how is thus indispensable. In comparison, the nCounter® system has the advantage of the specially developed nSolver™ software. The generated raw data can be imported into this software, normalized and analyzed by the user without any previous bioinformatic expertise.

### 4.3 Biological interpretation of transcriptome differences between the tumor entities studied

This technical study was based on two comparisons between contrasting tumor entities, the first between early and late stages of the spontaneously regressing canine cutaneous histiocytoma (CCH) and the second between two common canine perianal tumors with very distinct biological behaviors. The widely overlapping data obtained from the two methodological approaches clearly allowed for several oncologically interesting implications, with mutual confirmations by the two methods used. These results and interpretations, however, are subject of separate publications already published (36) or in preparation.

## 5 Conclusion

Taken together, our comparison between gene expression data obtained from QuantSeq 3′ and the nCounter® Canine IO Panel revealed very strong to moderately strong correlations when FFPE archival tissues stored up to 8 years long were used. The best correlations were achieved on the sample-wise level in both biological comparisons. Based on these strong correlations, it appears feasible to use either of the approaches to validate data generated by the other. nCounter® seems to be superior for validation compared to PCR for fragmented mRNA from FFPE tissue. There are, however, several more strategical and practical differences between the technologies and their actual use depends on the project’s study goal and design (Table 1). Clearly, both approaches make archival tissue well accessible for transcriptome studies in veterinary and comparative medicine. In particular, studies on rare entities or elusive biological cases will profit immensely from recent technological progress when compared to previous transcriptome methodologies.

## Data Availability

The raw data underlying the results presented in this study are publicly available from the Gene Expression Omnibus (GEO) database under the accession numbers GSE262020 (nCounter® data for CCH samples), GSE261791 (nCounter® data for HGA and AGASAC samples), GSE262022 (QuantSeq 3’ data for CCH samples), and GSE261790 (QuantSeq 3’ data for HGA and AGASAC samples. The source code and processed datasets are available on figshare: dx.doi.org/10.6084/m9.figshare.25768587.
